# Compression Modulus and Apparent Density of Polymeric Excipients during Compression—Impact on Tabletability

**DOI:** 10.3390/pharmaceutics14050913

**Published:** 2022-04-22

**Authors:** Barbara V. Schönfeld, Ulrich Westedt, Karl G. Wagner

**Affiliations:** 1 AbbVie Deutschland GmbH & Co. KG, Knollstraße 50, 67061 Ludwigshafen am Rhein, Germany; barbara.schoenfeld@abbvie.com (B.V.S.); ulrich.westedt@abbvie.com (U.W.); 2 Department of Pharmaceutical Technology, University of Bonn, Gerhard-Domagk-Straße 3, 53121 Bonn, Germany

**Keywords:** compression analysis, compaction behavior, density under pressure, particle density, elastic recovery, Heckel analysis, yield pressure, polymers

## Abstract

The present study focuses on the compaction behavior of polymeric excipients during compression in comparison to nonpolymeric excipients and its consequences on commonly used Heckel analysis. Compression analysis at compaction pressures (CPs) from 50 to 500 MPa was performed using a compaction simulator. This study demonstrates that the particle density, measured via helium pycnometer (ρ_par_), of polymeric excipients (Kollidon^®^VA64, Soluplus^®^, AQOAT^®^AS-MMP, Starch1500^®^, Avicel^®^PH101) was already exceeded at low CPs (<200 MPa), whereas the ρ_par_ was either never reached for brittle fillers such as DI-CAFOS^®^A60 and tricalcium citrate or exceeded at CPs above 350 MPa (FlowLac^®^100, Pearlitol^®^100SD). We hypothesized that the threshold for exceeding ρ_par_ is linked with predominantly elastic deformation. This was confirmed by the start of linear increase in elastic recovery in-die (ER_in-die_) with exceeding particle density, and in addition, by the applicability in calculating the elastic modulus via the equation of the linear increase in ER_in-die_. Last, the evaluation of “density under pressure” as an alternative to the ρ_par_ for Heckel analysis showed comparable conclusions for compression behavior based on the calculated yield pressures. However, the applicability of Heckel analysis for polymeric excipients was questioned in principle. In conclusion, the knowledge of the threshold provides guidance for the selection of suitable excipients in the formulation development to mitigate the risk of tablet defects related to stored elastic energy, such as capping and lamination.

## 1. Introduction

Tablets are the most preferred dosage form in pharmaceutical development showing vast benefits such as high-precision dosing, manufacturing efficiency, stability, and patient compliance [1,2]. Because of their poor aqueous solubility, many drug substances in the pipeline need to be formulated via enabling technologies [3]. One enabling formulation approach commonly used in the pharmaceutical industry is the amorphous solid dispersion approach, where the active pharmaceutical ingredient (API) is molecularly dispersed in a polymeric matrix in its amorphous form to enhance solubility. Technologies used for the ASD manufacture are hot-melt extrusion, an example of a fusion-based method, or spray drying as a solvent-based method [3]. In hot-melt extrusion, thermal and mechanical energy from corotating screws and heated barrels, followed by cooling, is used to produce the solid dispersion [4], which is further downstream processed to powder (milled extrudate) via a milling step. The latter step is important as dosage forms generated from the pure melt (e.g., injection molded or calendering) often result in slow dissolution without disintegration, which can be improved by mixing the ASD powder with tableting excipients [5]. Commonly used matrix polymers suitable for hot-melt extrusion are polyvinylpyrrolidone (PVP), polyvinylpyrrolidone/vinyl acetate (PVPVA, copovidone), polymethacrylates, hydroxypropyl methylcellulose (HPMC) or hydroxypropyl methylcellulose acetate succinate (HPMCAS), and polyvinyl caprolactam-polyvinyl acetate-polyethylene glycol graft copolymer (Soluplus^®^) [6]. Nevertheless, the final dosage form of ASDs-based drug products is mostly tablets, as demonstrated by recently marketed products [3,6].

Consequently, the compression of the resulting intermediates into tablets remains a key unit operation in the manufacturing process chain of a drug product. For that, tableting excipients such as fillers or binders are added to the formulation composition. The compression process as such can be generally divided into two stages. First is slippage and particle rearrangement, resulting in a volume reduction in the powder and denser packing structure [7]. Second is the subsequent reduction in the volume via applying higher compression forces and is associated with changes in the dimensions of the particles themselves, either by irreversible plastic deformation, reversible elastic deformation, or particle fragmentation into smaller particles [8,9]. However, during the compression of a powder, all deformation mechanisms can be present at different stages of the compaction process or can occur even simultaneously. A high proportion of elastic deformation during compression leads to the reduced mechanical strength of the tablets and probably to tablet defects such as capping or lamination [10]. In addition, even subsequent processing steps such as coating might be impacted by defects induced by the stored elastic energy within the tablets [11]. Busignies et al. [12] stated the importance of knowledge of elastic deformation to manufacture bilayer tablets.

Tableting excipients are usually categorized with respect to their main bonding mechanism and compaction behavior during compression, distinguishing between plastic or brittle deformation [9]. However, not only tableting excipients in the outer phase but also the main component of the intermediate, e.g., the matrix polymer of an amorphous solid dispersion, might profoundly affect the compaction behavior of the formulation. Thus, the selection of suitable excipients is crucial in the formulation development of solid dosage forms [13]. 

Compression analysis provides a deeper understanding of the compaction behavior of a powder under pressure, e.g., plasticity and elasticity. Common approaches were developed by Heckel, Kawakita and Lüdde, Kuentz and Leuenberger [14,15,16]. The Heckel equation considers the porosity of the tablet (either in-die or out-of-die) and the main compression force by using the force-displacement profile. It assumes that volume reduction by plastic deformation follows a first-order kinetic. In addition, it offers the opportunity to determine elastic recovery of the compact in-die, also known as fast elastic recovery, considering the decompression part of the Heckel plot. 

However, limitations of the Heckel analysis were observed in several studies [17,18]. The yield pressure (P_Y_), which represents the plasticity of the material, was found to be dependent on tableting parameters such as compression speed, tooling dimensions, as well as errors in porosity and pressure data used for calculation and elastic deformation [19,20,21]. Ilić et al. [22] observed differences in Heckel analysis comparing the “in-die” and the “out-of-die” method, stating the larger extent of error for the “out-of-die” method was due to elastic deformation. In addition, Sun, Grant [21] stated that Heckel analysis should not be considered for classification if the solid fraction of the powder during the compaction process is above 0.95. Apart from this, it was observed that the yield pressure values are lower for powders showing elastic deformation. Similar observations were made by Schlack [23], showing that starch undergoes solid-state compression and that the particle density should be corrected accordingly to ensure valid Heckel results. Heckel plots showing bending above a certain compaction pressure were likewise observed by Wünsch et al. [24]. Accordingly, Krumme et al. [25] introduced the “true density by compression”, which should be used for Heckel calculations instead of the particle density. The difference between particle density and “true density by compression” was considered most profound for Starch1500^®^ as elastic material and moderate for lactose as a brittle material. In conclusion, limitations for using the particle density for porosity calculations in compaction analysis were observed. 

In general, several studies investigated commonly used excipients for direct compression [26,27,28,29].

However, the difference in compaction behavior between polymeric and nonpolymeric materials, focusing on the apparent density in-die at different compaction pressures, has not been investigated before, especially not in correlation with the respective particle densities and the elastic recoveries (in-die). In this study, we hypothesize that the energy, which is needed to compress excipients (especially polymeric ones) beyond their particle density, correlates with predominantly elastic deformation and thus, can be measured as an increase in fast elastic recovery.

Therefore, the present study comprises the following aspects:

- Compaction analysis of **nonpolymeric tableting excipients** (di-calcium phosphate (DI-CAFOS^®^A60), tricalcium citrate tetrahydrate, spray-dried lactose monohydrate (FlowLac^®^100), mannitol (Pearlitol^®^100SD)) as well as common **polymeric tableting excipients** (partially pregelatinized maize starch (Starch1500^®^), microcrystalline cellulose (Avicel^®^PH101)) and **amorphous solid dispersion excipients** (copovidone (Kollidon^®^VA64), polyvinyl caprolactam-polyvinyl acetate-polyethylene glycol graft copolymer (Soluplus^®^), and hydroxypropyl methylcellulose (AQOAT^®^AS-MMP)). The excipients investigated are commonly used excipients in the development of solid dosage forms [28] and were chosen as targets to include excipients with different compaction behaviors (plastic, brittle) to ensure a comprehensive evaluation. The ASD excipients are matrix polymers used in the recently marketed drug products and are thus, of high importance [3];
- Assessment of an ASD manufactured via hot-melt extrusion consisting of ritonavir, copovidone, and sorbitan monolaurate and its respective tablet blend to evaluate the general impact on compaction analysis for ASDs;
- Discussion of consequences for commonly used Heckel analysis and the use of the “density under pressure” (500 MPa, dwell time 10 s) instead of particle density.

## 2. Materials and Methods

### 2.1. Materials

Di-calcium phosphate (DI-CAFOS^®^A60) was obtained from Chemische Fabrik Budenheim (Budenheim, Germany), tricalcium citrate tetrahydrate (TriCaCi) from Jungbunzlauer Ladenburg GmbH (Ladenburg, Germany), microcrystalline cellulose (Avicel^®^PH101) from FMC (Philadelphia, PA, USA), alpha-lactose monohydrate (FlowLac^®^100) from Meggle Group (Wasserburg, Germany), mannitol (Pearlitol^®^100SD) from Roquette GmbH (Frankfurt a. M., Germany), copovidone (polyvinylpyrrolidone-vinyl acetate copolymer, Kollidon^®^VA 64) and polyvinyl caprolactam-polyvinyl acetate-polyethylene glycol graft copolymer (Soluplus^®^) from BASF SE (Ludwigshafen, Germany), hypromellose acetate succinate (HPMCAS, AQOAT^®^AS-MMP) from Shin-Etsu Chemical Co. Ltd. (Tokyo, Japan), and partially pregelatinized maize starch (Starch1500^®^) from Colorcon Limited (Kent, UK). 

DI-CAFOS^®^A60 are aggregates of fine, almost spherical particles with an uneven surface and a d_50_ value of 60 µm [30]. TriCaCi is a powder of almost spherically shaped large agglomerates with a mean particle size of 135 µm [26]. Avicel^®^PH101 consists of irregularly shaped particles with a broad particle size distribution and a mean particle size of approximately 56 µm [31,32]. FlowLac^®^100 is manufactured via spray drying, which explains the spherical shape of the particles with a mean particle size of 110 µm [33]. In addition, Pearlitol^®^100SD is prepared via spray drying resulting in spherical particles with a mean particle size of 100 µm [34]. The mean particle size of Kollidon^®^VA 64 is 82 µm, and the particles are hollow spheres in a significant proportion [35]. Soluplus^®^ appears as particles with a diameter of approximately 340 microns in a mostly spherical shape, according to the technical information of the vendor [36]. AQOAT^®^AS-MMP consists of particles with a mean particle size of approximately 300 µm. Starch1500^®^ consists of particles with a broad particle size distribution and a mean particle size of approximately 65 µm [37]. 

The amorphous solid dispersion (ASD) consists of 15% (*w*/*w*) ritonavir, 74% (*w*/*w*) copovidone, 10% (*w*/*w*) sorbitan monolaurate, and 1% (*w*/*w*) silicon dioxide, and the tablet blend of 87.1% (*w*/*w*) milled extrudate (ASD), 11.7% (*w*/*w*) di-calcium phosphate, 0.9% (*w*/*w*) silicon dioxide, and 0.3% (*w*/*w*) sodium stearyl fumarate. Both ASD and tablet blend composition were in accordance with the marketed Norvir^®^ formulation serving as model ASD formulation in the present study representative for other ASDs. The ASD was manufactured by hot-melt extrusion and was kindly provided by AbbVie Deutschland GmbH & Co. KG, Ludwigshafen, Germany. 

In detail, ritonavir (purity > 99.8%) was obtained from AbbVie Inc. (North Chicago, IL, USA) and sorbitan monolaurate (Span20^®^) from CRODA (Nettetal, Germany). Di-calcium phosphate (DI-CAFOS^®^ A60) was purchased from Chemische Fabrik Budenheim (Budenheim, Germany), fumed silicon dioxide (Aerosil^®^200) from Evonik Industries (Essen, Germany), and sodium stearyl fumarate (PRUV^®^) from JRS Pharma (Rosenberg, Germany).

To ease the readability of the figures within the article, the respective excipients were displayed without their trademarks.

### 2.2. Methods

#### 2.2.1. Hot-Melt Extrusion (HME)

Hot-melt extrusion was performed on a commercial scale corotating twin-screw extruder (ZSK 58, Coperion GmbH, Stuttgart, Germany) combined with a calender (COLLIN Lab & Pilot Solutions GmbH, Maitenbeth, Germany) equipped with ellipsoidal-shaped molds to obtain the ritonavir containing amorphous solid dispersion in the form of extrudate beads. The extrusion parameters were as follows: temperature profile 20/80/100/110 °C, screw speed 185 rpm, and vacuum 150 mbar. The extrudate beads were milled at 7000 rpm using an impact mill (Alpine UPZ100, Hosokawa Alpine, Augsburg, Germany) equipped with a 1.3 mm sieve.

#### 2.2.2. Tablet Blend Preparation

Outer phase excipients were added to the milled extrudate (87.1% (*w*/*w*)) consisting of ritonavir as a drug substance according to the Norvir^®^ formulation, an antiretroviral drug product (tablet) used in combination with other medications to treat the human immunodeficiency virus infection (HIV) and acquired immunodeficiency syndrome (AIDS): di-calcium phosphate as a filler (11.7% (*w*/*w*)), fumed silicon dioxide as a glidant (0.9% (*w*/*w*)), and sodium stearyl fumarate (0.3% (*w*/*w*)) as a lubricant. The tablet blend was prepared using a bin blender (Bohle PM400, L.B. Bohle Maschinen + Verfahren GmbH, Enningerloh, Germany), and a screening machine (Bohle BTS, L.B. Bohle Maschinen + Verfahren GmbH, Enningerloh, Germany) with a 1.5 mm mesh by the following consecutive steps: (1) sieving, and (2) blending for 9 min at 6 rpm. 

The milled extrudate is named in the following as HME ASD and the respective tablet blend as HME TB.

#### 2.2.3. Particle Density (Pycnometric Density, ρ_par_)

Particle (pycnometric) density was determined using a helium pycnometer (AccuPyc 1340, Micromeritics GmbH, Aachen, Germany). The helium pycnometer was equipped with a 10 cm^3^ sample chamber and was operated at a cycle fill pressure of 134.45 kPa and an equilibration rate of 0.0345 kPa/min. Purging of the sample chamber was conducted 10 times prior to the measurement. For each analysis, 5 cycles were performed. All samples were measured as triplicates.

#### 2.2.4. Density under Pressure (ρ_pre_)

The density under pressure (ρ_pre_) was determined according to Krumme et al. [25] using a single punch compression simulator (HB-50, Huxley Bertram Engineering Limited, Cambridge, UK) equipped with 10 mm round, flat face tooling. A compaction profile was designed to ensure proper air release during compaction and maximum densification, including a ramp of 20 s up to the maximum compaction pressure of 500 MPa and a dwell time of 10 s. For each respective material, 6 tablets were manufactured to determine the density under compaction pressure, which was used for further calculations.

#### 2.2.5. Density Ratio

The density ratio ρ_ratio_ between the density under pressure (ρ_pre_) and particle density (ρ_par_) was calculated for comparison reasons according to the following equation (Equation (1)):

(1) $$\rho_{\mathrm{ratio}}\left[\%\right]=\frac{\rho_{\mathrm{pre}}-{\;\rho }_{\mathrm{par}}}{{\;\rho }_{\mathrm{par}}}\times 100$$

#### 2.2.6. Compression Analysis

A single punch compression simulator (HB-50, Huxley Bertram Engineering Limited, Cambridge, UK) equipped with 10 mm round, flat face tooling was used for compression analysis. Tablets (*n* = 6) targeting a mass of 200 mg (400 mg for DI-CAFOS A60 due to the high bulk density) were manufactured at ten compaction pressures ranging from 50 MPa to 500 MPa. The production scale tablet press Fette 3090i (61 stations, Euro-B tooling) at a turret speed of 15 rpm (according to a linear speed of 0.32 m/s and dwell time of 19 ms) was simulated. Tablets were characterized regarding tablet weight (analytical balance, Sartorius BP 61 S 0CE, Sartorius AG, Goettingen, Germany), thickness, and diameter (caliper, Hommel Hercules Werkzeughandel GmbH & Co KG, Viernheim, Germany), and breaking force (Lab-line H4, Kraemer Elektronik GmbH, Darmstadt, Germany).

#### 2.2.7. Compaction Pressure

The compaction pressure (CP in N/mm^2^ or MPa) was calculated from the applied main compression force and cross-sectional area of the punch (Equation (2)) [38].

(2) $$\mathrm{CP}=\frac{\mathrm{Main}\;\mathrm{Compression}\;\mathrm{Force}\;\left[N\right]}{\mathrm{Cross}-\mathrm{sectional}\;\mathrm{Area}\;\left[{\mathrm{mm}}^{2}\right]}$$

#### 2.2.8. Apparent Density (In-Die)

The apparent density of the tablet in-die (ρ_app_) was calculated from the tablet mass (m) divided by the volume of the tablet at minimal punch separation (V_minSP_) (Equation (3)):

(3) $$\rho_{\mathrm{app}}=\frac{m}{V_{\mathrm{minSP}}\;}$$

#### 2.2.9. Particle Density Threshold

The particle density threshold is defined as the value where the apparent density exceeds the particle density. For determination, the apparent density results (mean values) were plotted depending on the compaction pressure applied (mean values) and exponentially fitted (one-phase decay). The equation for the exponential fit function was used to calculate the respective particle density threshold compaction pressure (single value). 

#### 2.2.10. Solid Fraction (In-Die)

Solid fraction in-die (SF) is the apparent density of the tablet in-die (ρ_app_) divided by the particle (pycnometric) density (ρ_par_) of the powder (Equation (4)) [38]:

(4) $$\mathrm{SF}=\frac{\rho_{\mathrm{app}}}{\rho_{\mathrm{par}}}$$

#### 2.2.11. Elastic Recovery (In-Die)

The elastic recovery in-die (ER_in-die_) is calculated as follows (Equation (5)):

(5) $${\mathrm{ER}}_{\mathrm{in}-\mathrm{die}}\left[\%\right]=\frac{V_{\mathrm{minCP}}-V_{\mathrm{minPS}}}{V_{\mathrm{minCP}}}\times 100$$

where V_minCP_ is the tablet volume at minimal compaction pressure (minCP) after the compression process, and V_minPS_ is the tablet volume at the minimal punch separation (minPS) (=minimal in-die tablet volume).

#### 2.2.12. Elastic Modulus (Young’s Modulus)

The elastic modulus displays the resistance of a material to being elastically deformed when mechanical stress is applied and can be determined via the slope of its stress–strain curve. Stiffer materials have higher elastic moduli compared to elastic materials.

The elastic modulus E is defined by the following equation (Equation (6)):

(6) $$E=\frac{F}{A}\;\frac{L_{0}}{∆L}$$

where F is the force applied to the surface A (“stress”), L_0_ is the initial length of a solid object, and ∆L is the reduction in the length (“longitudinal strain”).

ER_in-die_ is calculated by the difference in tablet dimensions under pressure (stress) which corresponds to the reciprocal of the strain (L_0_/∆L). Since a linear increase in ER_in-die_ was observed after the exceedance of the particle density, the slope of the linear equation ($a_{{\mathrm{ER}}_{\mathrm{eq}}})$ was used as a constant to determine the elastic modulus E_mod_ (Equation (7)).

(7) $$E_{\mathrm{mod}}\left[\mathrm{MPa}\right]=\frac{1}{a_{{\mathrm{ER}}_{\mathrm{eq}}}}\times 100$$

#### 2.2.13. Heckel Analysis (In-Die)

**Figure 1:**
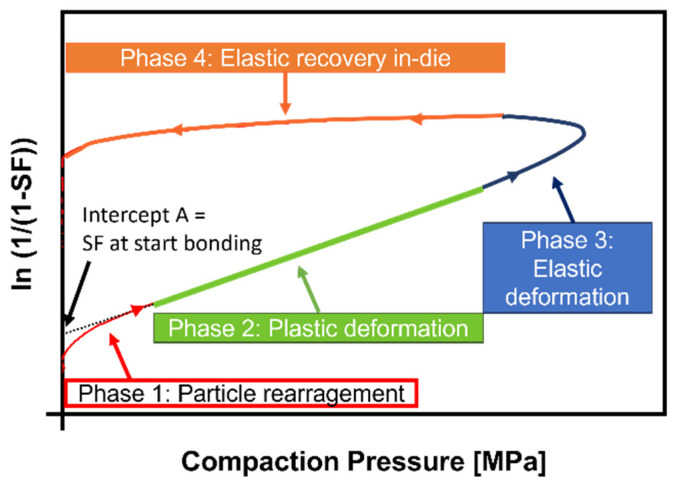
Schematic Heckel plot in-die.

The compression behavior in terms of deformation was studied by means of the Heckel Equation (8) as the “in-die method” [14]. The Heckel equation assumes that volume reduction by plastic deformation follows a first-order kinetic:

(8) $$\ln\left(\frac{1}{1-\mathrm{SF}}\right)=\;k\;\times \mathrm{CP}+A$$

where CP is the compaction pressure, and SF is the solid fraction (relative density) at CP. Slope k and intercept A of the linear ascending part of the Heckel plot (phase 2, plastic deformation phase) are material-dependent constants. The SF was calculated as the “in-die method” considering the punch gap during compression for tablet volume calculation. However, instead of just using the particle density for SF calculation, the “density under compaction pressure” (ρ_pre_) was additionally used in accordance with Krumme et al. [25]. For comparison reasons, Heckel plots at 100 and 300 MPa were generated by using (a) the particle density (ρ_par_) and (b) the density under pressure (ρ_pre_) (*n* = 6). Figure 1 shows a typical Heckel plot with stages of powder densification during compaction: (1) particle rearrangement, (2) plastic deformation, and (3) elastic deformation. After the maximum compaction pressure is applied, the maximum material densification is reached with a short delay before the last phase, (4) elastic recovery in-die occurs, resulting in a less dense compact at the end of the compaction process. 

The linear section of the ascending part of the Heckel plot corresponds to phase 2 (Figure 1) and was selected via the best correlation coefficient (R^2^ > 0.999) for the linear regression using Igor Pro v8 (WaveMetrics Inc., Lake Oswego, OR, USA). The range for the region was set to 30 MPa for the Heckel plots at 100 MPa compaction pressure (CP) and 100 MPa for those at 300 MPa.

The yield pressure (P_Y_) was calculated as reciprocal of k (slope of the linear part) and is inversely correlated to the start of plastic deformation (Equation (9)) [14]:

(9) $$P_{Y}=\frac{1\;}{k}$$

## 3. Results

### 3.1. Density Ratio: Particle Density vs. Density under Pressure

**Figure 2:**
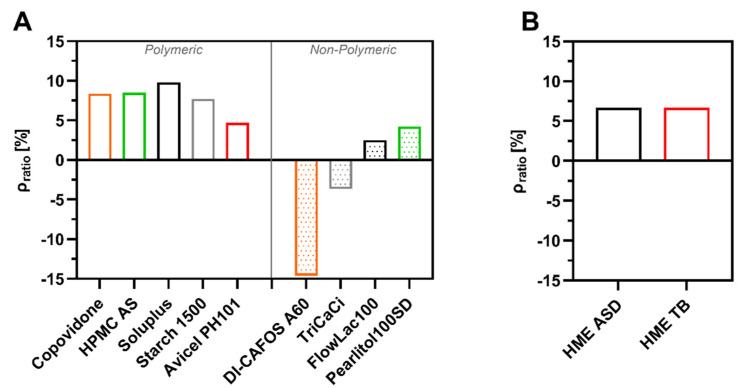
Density ratio of (**A**) all excipients tested and (**B**) HME ASD and HME TB.

The results for density (ρ_pre_ and ρ_par_) and density ratio (ρ_ratio_) are shown in Table 1**.** and visualized in Figure 2. The density under pressure values were higher compared with the particle density values determined via helium pycnometer resulting in positive density ratio values in all cases except for DI-CAFOS^®^A60 and TriCaCi (Figure 2A). Polymeric fillers or matrix polymers for ASD manufacture showed the highest positive density ratio values, e.g., Starch1500 with 7.7% or Soluplus^®^ with 9.8%. Brittle fillers such as TriCaCi (−3.7%) and DI-CAFOS^®^A60 (−14.6%) showed negative density ratio results. HME ASD and HME TB, both based on copovidone as matrix polymer, showed comparable density ratio values (6.7%) regardless of the addition of outer phase excipients in accordance with the density ratio value of pure copovidone (8.4%) (Figure 2B).

### 3.2. Apparent Density In-Die vs. Compaction Pressure

**Figure 3:**
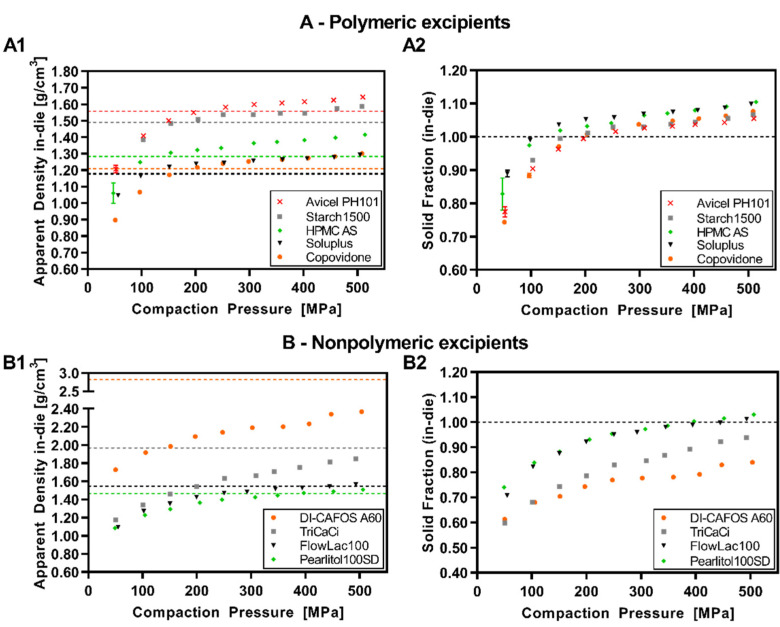
(**A**) Polymeric excipients: apparent density in-die vs. compaction pressure (dotted lines = respective particle densities) (**A1**); solid fraction in-die vs. compaction pressure (**A2**); (**B**) Nonpolymeric excipients: apparent density in-die vs. compaction pressure (dotted lines = respective particle densities) (**B1**); solid fraction in-die vs. compaction pressure (**B2**).

Figure 3 shows the apparent density values of the tablets at minimum punch separation during compression at different compaction pressures (A1 = polymeric excipients; B1 = nonpolymeric) and the respective particle densities as dotted lines. For better interpretability, a normalized visualization via solid fraction in-die is shown in Figure 3A2 for polymeric excipients and in Figure 3B2 for nonpolymeric. The data indicated an increase in apparent density/solid fraction with increasing compaction pressure reaching a plateau at high compaction pressures. It was observed that the respective particle density values were exceeded for all polymeric excipients at the latest above a compaction pressure of 200 MPa, resulting in SF values above 1. For FlowLac^®^100 and Pearlitol^®^100SD as nonpolymeric excipients, the corresponding threshold value was at higher compaction pressures (450–500 MPa), whereas neither DI-CAFOS^®^A60 nor TriCaCi exceeded the particle density during compression, up to compaction pressures of 500 MPa. Considering the trend of the values, the particle density will probably not be exceeded even at higher CPs. Notably, TriCaCi showed higher SF values compared to Di-CAFOS^®^A60, which might be related to particle morphology. TriCaCi consists of larger agglomerates of lower micron to a submicron particle size which might shift more easily into denser structures during compression, resulting in high interaction forces and, thus, strong compacts as observed by Hagelstein et al. [27]. 

**Figure 4:**
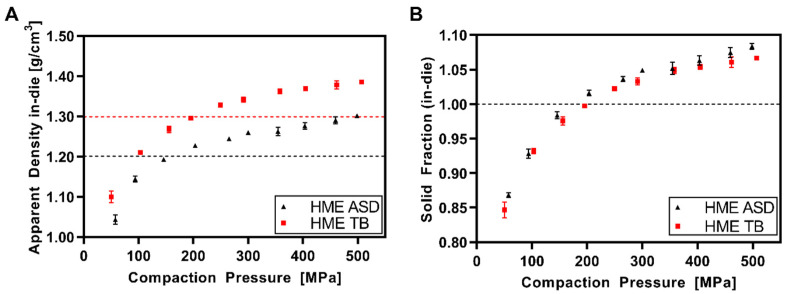
HME ASD and HME TB: (**A**) apparent density in-die vs. compaction pressure (dotted lines = respective particle densities); (**B**) solid fraction in-die vs. compaction pressure.

Figure 4 displays the apparent density/solid fraction in-die data for the milled extrudate (HME ASD) and the respective tablet blend (HME TB) based on copovidone as the matrix polymer. The trend was in accordance with the data for pure copovidone: the apparent density plateau was at around 1.3 g/cm^3^. The threshold for exceeding the particle density was in a comparable range (HME ASD: 159 MPa; HME TB:196 MPa; copovidone: 188 MPa). However, a clear shift for the HME TB threshold to higher compaction pressure could be observed. This might be explained by the addition of 11.7% (*w*/*w*) DI-CAFOS^®^A60 showing no exceedance of the particle density in the investigated pressure range. Besides, the drug substance ritonavir and the surfactant sorbitan monolaurate lowered the threshold of the ASD compared to pure copovidone. 

### 3.3. Elastic Recovery In-Die vs. Compaction Pressure

**Figure 5:**
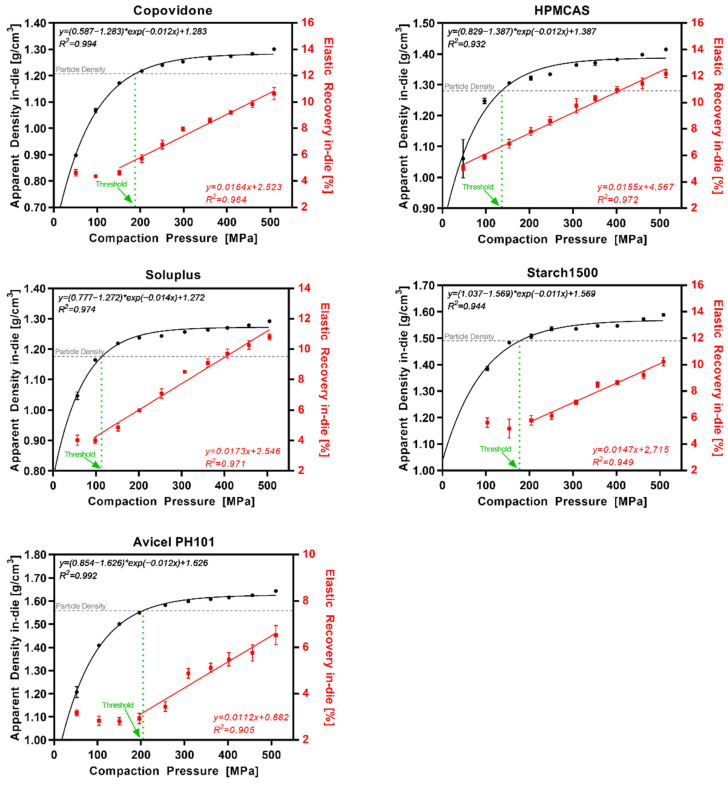
Polymeric excipients: apparent density in-die (black) and elastic recovery in-die (red) at different compaction pressures; particle density (grey dotted line); threshold in CP where ρ_app_ = ρ_par_ (green dotted line).

**Figure 6:**
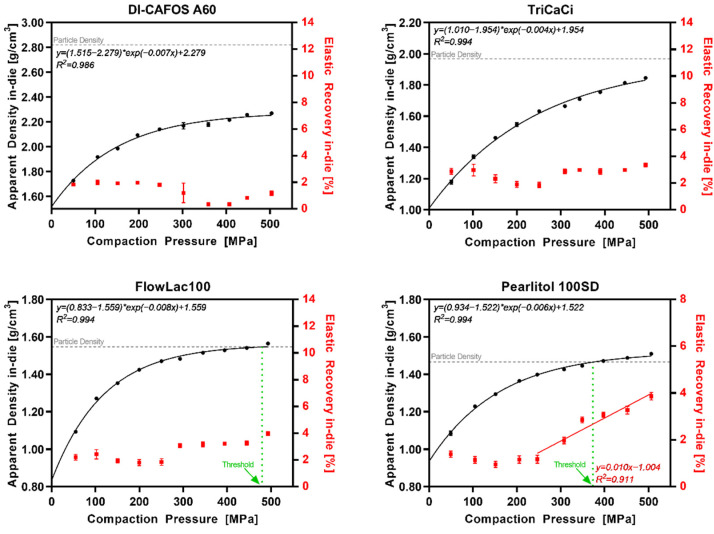
Nonpolymeric excipients: apparent density in-die (black) and elastic recovery in-die (red) at different compaction pressures; particle density (grey dotted line); threshold in CP where ρ_app_ = ρ_par_ (green dotted line).

The interplay of the apparent density and the elastic recovery in-die (ER_in-die_) at different CPs for polymeric excipients is shown in Figure 5 and for nonpolymeric excipients in Figure 6. Polymeric excipients exhibited a linear increase (R^2^ > 0.90) in ER_in-die_ with increasing CPs. Interestingly, the increase in ER_in-die_ started at about the same CP as the threshold for exceedance of the particle density during compression. The nonpolymeric excipients DI-CAFOS^®^A60, TriCaCi, and FlowLac^®^100 showed no increase in ER_in-die_ with increasing CPs up to 500 MPa, whereas Pearlitol^®^100SD showed an increase starting at 250–300 MPa. Overall, the ER_in-die_ values were higher for polymeric excipients even at the start (4–6%) compared with nonpolymeric excipients (<4%).

**Figure 7:**
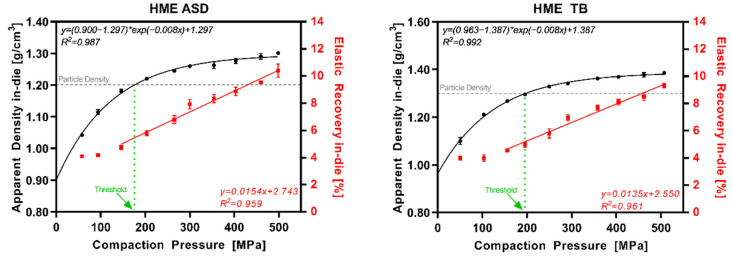
HME ASD and HME TB: apparent density in-die (black) and elastic recovery in-die (red) at different compaction pressures; particle density (grey dotted line); threshold in CP where ρ_app_ = ρ_par_ (green dotted line).

Figure 7 displays the results for the HME ASD and HME TB representing ASD formulations in general. Additionally, in this case, the data were in accordance with the pure copovidone data set. The ER_in-die_ increased above 150–200 MPa following linear regression with a R^2^ above 0.95.

### 3.4. Particle Density Threshold 

**Figure 8:**
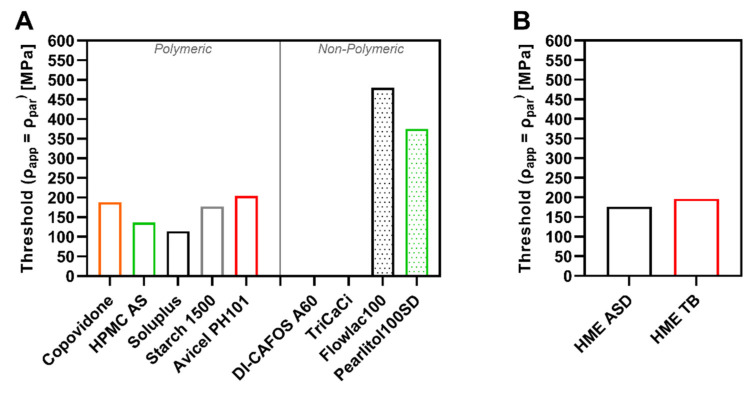
Particle density threshold in CP (ρ_app_ = ρ_par_) of (**A**) polymeric and nonpolymeric excipients, and (**B**) of HME ASD and TB.

The thresholds in CP of the investigated excipients (polymeric and nonpolymeric) for the exceedance of the particle density during compression (ρ_app_ = ρ_par_) are visualized in Figure 8A, whereas the thresholds for the HME ASD and HME TB in Figure 8B. The threshold simultaneously indicated the start of a linear increase in elastic recovery (in-die). The threshold values for polymeric excipients were between 100–200 MPa, and thus, the particle density was already exceeded at low CPs. In contrast, the threshold values for nonpolymeric values were either above 350 MPa (FlowLac^®^ and Pearlitol^®^100SD) or never reached (DI-CAFOS^®^A60, TriCaCi).

### 3.5. Elastic Modulus (Young’s Modulus, E_mod_)

Table 2 summarizes the elastic modulus (E_mod_) values calculated based on the slope of the linear regression equation for ER_in-die_. E_mod_ values for the polymeric excipients were in the range of 5.8–8.9 GPa. E_mod_ values for HME ASD (6.5 GPa) and HME TB (7.4 GPa) were similar to pure copovidone (6.1 GPa). However, the HME TB E_mod_ value was higher compared with the ASD, assuming higher stiffness of the material, which might be related to DI-CAFOS^®^A60, known as brittle filler. 

E_mod_ values for nonpolymeric excipients could not be calculated via this approach since no linear increase in ER_in-die_ was observed for those excipients, except for Pearlitol^®^100SD. An E_mod_ for Pearlitol^®^100SD of 10.2 GPa was higher compared to nonpolymeric excipients indicating a slightly higher degree of stiffness.

### 3.6. Heckel Analysis (In-Die)

**Figure 9:**
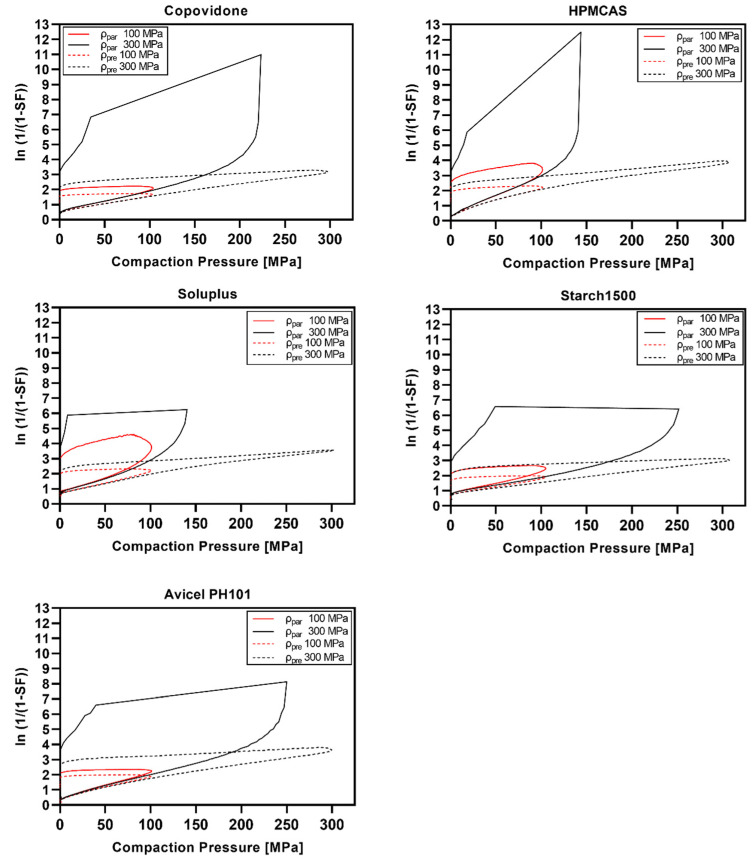
Heckel plots of polymeric excipients at 100 and 300 MPa calculated using particle density (ρ_par_) or density under pressure (ρ_pre_).

**Figure 10:**
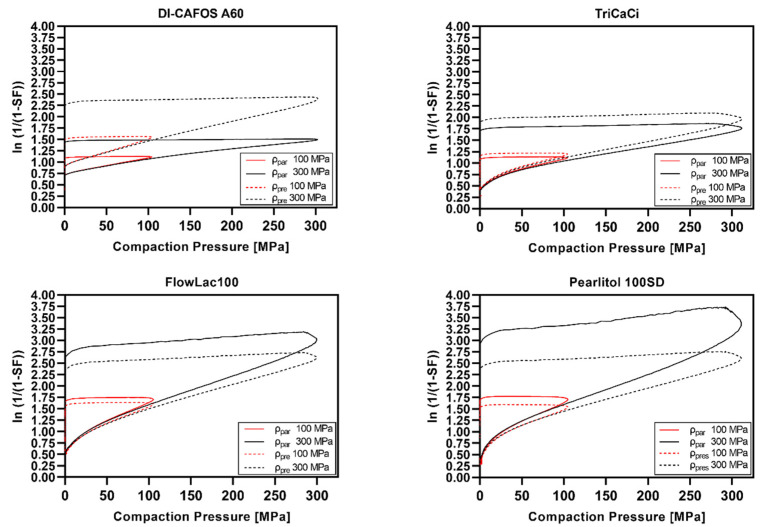
Heckel plots of nonpolymeric excipients at 100 and 300 MPa calculated using particle density (ρ_par_) or density under pressure (ρ_pre_).

**Figure 11:**
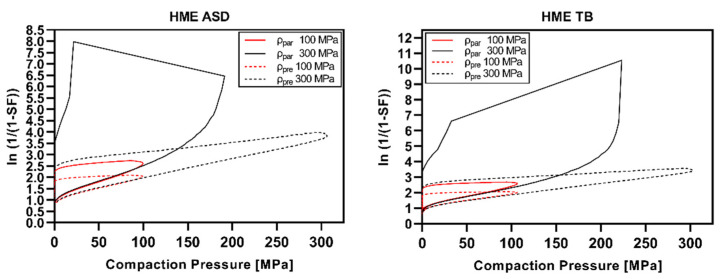
Heckel plots of HME ASD and HME TB at 100 and 300 MPa calculated using particle density (ρ_par_) or density under pressure (ρ_pre_).

The Heckel plots are visualized in Figure 9 (polymeric excipients), Figure 10 (nonpolymeric excipients), and Figure 11 (HME ASD and HME TB). Heckel plots could not be generated properly for CPs of 300 MPa for polymeric excipients (Figure 9) and the ASD model formulations (Figure 11) if calculated with the particle density. The particle density was exceeded at compaction pressures of 100–200 MPa, resulting in SF values above 1, i.e., an invalid Heckel model. As mentioned in Section 2.2.10, the density under CP (ρ_pre_) was additionally considered for Heckel analysis according to Krumme et al. [25], leading to valid Heckel plots even at CPs of 300 MPa. 

Overall, nonpolymeric excipients showed distinct curvature at the beginning of the Heckel plot related to particle rearrangement and fragmentation, which was much less pronounced for polymeric excipients. Moreover, polymeric excipients showed a larger extent of elastic recovery in the decompression phase compared with nonpolymeric excipients. 

The Heckel plots for HME ASD and TB (Figure 11) were widely comparable to the curve shape of pure copovidone (Figure 9).

The calculated results of the Heckel analysis (in-die) are summarized in Table 3 for CP of 100 MPa (CP100) and 300 MPa (CP300) for all excipients as well as HME ASD and HME TB. The calculation of mean yield pressure (PY) allows an interpretation of the start of plastic flow, whereas the SF at intercept A of the linear ascending part of the Heckel plot indicates where SF bonding would occur. The highest mean yield pressures (PY) were observed for the nonpolymeric excipients such as DI CAFOS®A60 (ρpar: 293 MPa at CP100; 585 MPa at CP300) and TriCaCi (ρpar: 243 MPa at CP100; 336 MPa at CP300;) as expected for brittle fillers. At CP100 (ρpar), FlowLac®100 (138 MPa) and Pearlitol®100SD (130 MPa) exhibited values above 100 MPa, indicating a lower degree of brittle deformation. 

In contrast, the polymeric excipients revealed P_Y_ values at CP100 (ρ_par_) below 100 MPa, indicating viscoelastic to plastic compaction behavior (HPMCAS: 42 MPa, Soluplus^®^: 57 MPa, copovidone: 80 MPa, Starch1500^®^: 82 MPa). The respective P_Y_ values for HME ASD (79 MPa) and TB (88 MPa) were similar to pure copovidone, as copovidone is a major component of the formulation. Still, slightly higher values at both CPs could be observed for the HME TB, which was likely attributed to the brittle filler DI-CAFOS^®^A60 in the blend.

**Figure 12:**
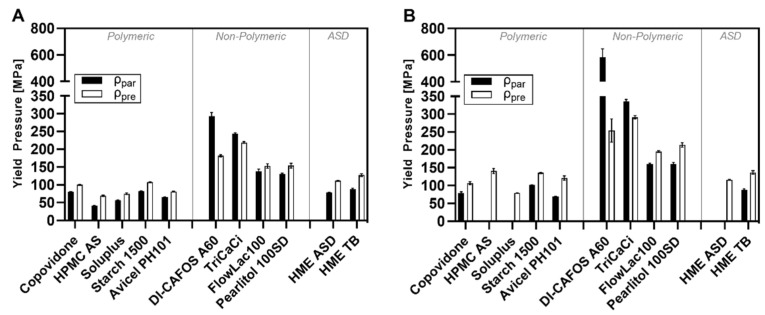
Yield pressure of excipients and HME ASD and HME TB at different compaction pressures (**A**) 100 MPa and (**B**) 300 MPa.

Figure 12 clearly visualizes the shift in yield pressures (P_Y_) depending on the density used for calculation. If the density ratio was positive (ρ_pre_ > ρ_par_) for the respective excipient, as seen for all polymeric excipients, the SF values were lower, resulting in a shift towards lower *Y*-axis values and lower slope values for the regression line in the Heckel plots. Thus, the yield pressures (P_Y_) calculated with ρ_pre_ were slightly higher compared with those calculated via ρ_par_. Exemplarily, the P_Y_ of HPMCAS at CP_100_ increased from 42 MPa to 69 MPa and the P_Y_ of Soluplus^®^ from 57 MPa to 75 MPa. Although a clear shift could be observed, the trend between the investigated excipients stayed the same. For instance, for polymeric excipients, the ranking at CP_100_ was as follows, independently from the density used for calculation: Starch1500^®^ > Copovidone (Kollidon^®^VA64) > Avicel^®^PH101 > Soluplus^®^ > HPMCAS (AQOAT^®^AS-MMP). Since the ρ_ratio_ for DI-CAFOS^®^A60 and TriCaCi was negative, the P_Y_ values calculated with ρ_pre_ (DI-CAFOS^®^A60: 181 MPa, TriCaCi: 219 MPa) were lower compared with those with ρ_par_ (DI-CAFOS^®^A60: 293 MPa, TriCaCi: 243 MPa). For the P_Y_ values at CP_300_ similar shift tendencies could be observed.

## 4. Discussion

### 4.1. Powder Density in Compression Analysis—Differences and Consequences

The importance of the correct particle density determination for compression analysis, which significantly influences the resulting compaction parameters such as yield pressure (P_Y_) within the Heckel model, has already been reported in the literature [18,21,23,25,39,40,41,42]. Gabaude et al. [42] clearly demonstrated that errors in measuring particle density have a greater effect on P_Y_ than the errors incurred from not correcting the displacement measurements due to punch elasticity. Thus, several publications focus on a suitable determination of the particle density considering the true conditions during compression analysis. For example, Sun [43] introduced a new method to determine the true density by calculating it via compaction data. This method involves the nonlinear regression of compaction pressure–tablet density data based on a modified Heckel equation. The intention is to avoid the impact of releasing water during the determination of the true density via helium pycnometer. Krumme et al. [25] introduced the “true density by compression” determined by compression experiments at a very high load level (0.73 GPa) under vacuum conditions. In most cases, the alternative approaches to determine the density used for compression analysis were observed to lead to higher absolute P_Y_ values. Krumme et al. [25] observed the strongest deviation from the pycnometric (true/particle) density for Starch1500^®^ assumed to be related to a high number of internal pores, whereas the deviation for lactose was much smaller. The present study corroborated these findings exceeding them to other polymeric excipients showing pronounced positive ρ_ratio_ values, whereas nonpolymeric excipients had “density under pressure” values below the particle density (negative ρ_ratio_) or less pronounced deviations from the ρ_par_. The density values (e.g., ρ_par_ vs. ρ_pre_) differ depending on the method used. This suggests that deriving compaction behavior parameters should be assessed relatively and not absolutely. Meaning, comparability between calculated values might only be given if the same method for density determination is used. Therefore, no clear boundaries for categories can be set considering all kinds of methods. Taking into account that even more variables such as simulated tablet press, tooling, compression speed, and applied force have an influence on compaction behavior parameters, the suggested approach seems reasonable.

### 4.2. Particle Density Exceeded during Compression and the Impact on Elastic Recovery

The present study observed exceeding particle densities for polymeric excipients and the amorphous solid dispersion (ASD) model formulation already at low CPs (e.g., Starch1500^®^ at 177 MPa and Avicel^®^PH101 at 204 MPa). Similar results have been reported by Van der Voort Maarschalk et al. [11] for pregelatinized potato starch, by Schlack [23] for Starch1500^®^, as well as for microcrystalline cellulose (Avicel^®^PH102) assuming solid-state compression. However, the absolute exceedance threshold was slightly higher, with values around 240 MPa for Starch1500^®^ and 280 MPa for Avicel^®^PH102. This might be related to differences in tablet press type (eccentric vs. rotary press) and dwell time differences. Accordingly, Wünsch et al. [24] showed in their recently published work that solid-state compression for microcrystalline cellulose (Vivapur^®^102) and paracetamol at CPs around 250–300 MPa and for lactose (anhydrous) at 400 MPa. Consequently, the bulk modulus measured by mercury porosimetry was used to characterize the deformation behavior of powders instead of ρ_par_.

The current study showed a clear difference in threshold values between polymeric and nonpolymeric excipients. In addition, processing polymeric excipients used for ASD manufacture via hot-melt extrusion led to a threshold value in a similar CP range which was exemplarily shown for copovidone. Consequently, it seems worthy to consider the threshold value in CP as a potential alternative approach to categorize materials based on their compaction behavior; excipients with threshold values below 300 MPa showed predominantly viscoelastic/plastic deformation, whereas excipients with values above 350 MPa or without any determinable threshold value exhibit brittle compaction behavior. However, Yost et al. [44] clearly demonstrated that for tablet formulation development, the API plays an important role in showing lesser suitability being classified by the common approaches. In addition, the results offer the opportunity to rate the risk for tablet defects based on stored elastic energy depending on the required compaction pressure for a certain mechanical strength of the tablet. However, considering tabletability, there are more factors involved in influencing the mechanical strength of the tablets, such as particle morphology, as demonstrated in previous work [45]. It was shown that tabletability as such is mostly influenced by the particle morphology comparing ASDs with similar solid-state by hot-melt extrusion, spray-drying, or vacuum drum drying. Besides, the threshold of increasing total elastic recovery was comparable for all investigated ASDs independent of the technology. 

Moreover, the present study indicated that exceeding the particle density might be linked with the start of a linear increase in fast (initial) elastic recovery (ER_in-die_), especially for polymeric excipients. Consequently, the energy applied to the compact over the threshold value was converted into elastic deformation of the material itself and not just of the particles. This energy was then released immediately during the in-die decompression phase. Thus, a solid-state compression can be suggested according to this data set. The demonstrated applicability of calculating the elastic modulus of a powder based on the linear increase in the ER_in-die_ above the particle density threshold confirmed this conclusion. In general, the absolute values for the elastic modulus depend on the determination method. However, similar trends could be observed using the method presented here compared to common literature. Iyer et al. [46] indicated that polymeric excipients such as copovidone (6.3 GPa), HPMCAS (3.0 GPa), or Avicel^®^PH101 (8.1 GPa) exhibit lower elastic modulus values corresponding to higher elasticity compared to nonpolymeric excipients such as dibasic calcium phosphate anhydrous (41 GPa) or lactose monohydrate (11.3 GPa) assuming higher degree in stiffness. E_mod_ values determined in the current study were in the range of 5–9 GPa (copovidone 6.1 GPa, Starch1500^®^ 6.8 GPa, Avicel^®^PH101 8.9 GPa) for polymeric excipients. Consequently, our results led to similar conclusions about elasticity/stiffness and thus, demonstrated proof of predominantly elastic deformation via solid-state compression at compaction pressures above the particle density threshold.

Similar conclusions were drawn by Christian [47] for Eudragit^®^ RS PO polymer used in sustained-release tablets. By comparing the in-die with the out-of-die porosity in dependency of the CP, it was observed by Christian [47] that the porosity of the tablets out-of-die was not changing, although the porosity in-die was being further reduced. The plateau was reached with exceeding particle density which was at CPs of about 150 MPa and in line with the values for polymeric excipients of the present study.

For nonpolymeric excipients, an increase in ER_in-die_ was not observed in the investigated CP range except for Pearlitol^®^100SD. However, Pearlitol^®^100SD exhibited a threshold in this CP range, explaining the observed increase in ER_in-die_. 

Overall, the extent of the ER_in-die_ for polymeric and nonpolymeric excipients was in accordance with the literature. Tanner [48] noted ER_in-die_ values for calcium phosphate grades of 1–5%, lactose grades of 1–6%, microcrystalline cellulose grades of 7–12%, HPMC of 14–17%, and starch grades of 14–18%. The present study determined values in similar ranges for nonpolymeric excipients, e.g., <4% for FlowLac^®^100 and <3% for DI-CAFOS^®^A60. For polymeric excipients, the values were slightly lower, as reported by Tanner [48] (HPMCAS: 5–13%, Starch1500^®^: 5–11%). Zhang et al. [49] observed higher ER_in-die_ values from 15–340 MPa for Avicel^®^PH101 (3–3.5%) as a polymeric excipient, whereas lactose monohydrate, mannitol, or dibasic calcium phosphate showed values around 1.5–2%. 

The elastic recovery caused by stored elastic energy is assumed to be one of the main causes of capping or lamination defects, according to van der Voort Maarschalk et al. [50]. Moreover, it is known to diminish the tensile strength of tablets by rupturing bonds between particles. Consequently, determining and considering the threshold for formulation and process development purposes might be useful to mitigate the risk of tablet defects related to stored elastic energy. It is certainly conceivable to include the threshold for exceedance of the particle density in a risk assessment for drug product development. 

### 4.3. Impact of Density Determination on Heckel Analysis

The Heckel plots of polymeric excipients indicated pronounced viscoelastic/plastic deformation based on the strong elastic recovery and the less distinct particle rearrangement/fragmentation phase. Nonpolymeric excipients instead showed a long particle rearrangement/fragmentation phase and much less elastic recovery. The shapes of the Heckel plots presented in the current study were in accordance with the literature [23,25,26]. 

However, the present work demonstrated that Heckel plots were not valid for CPs exceeding the threshold of particle density if the particle density is used for porosity calculation. Similar observations were made by Schlack [23] for Starch1500^®^ and Avicel^®^PH102. It was stated that Heckel plots could not be plotted properly above a CP of 250 MPa. Wünsch et al. [24] observed bending of the Heckel curve for microcrystalline cellulose and paracetamol above 250–300 MPa to high y-axis values assuming less suitability for Heckel analysis if using noncorrected density data. Additionally, Mahmoodi et al. [51] presented Heckel plots showing strong bending towards higher y-axis values in the ascending part already at CPs below 250 MPa for PEG 6000, maize starch, Starch1500^®^, PVP, and aspirin. Moreover, recently, Yost et al. [44] stated that Heckel results should be taken with caution for elastic materials.

The yield pressure (P_Y_) values calculated based on the linear regression within the ascending part of the Heckel plot were consistent with current literature [13,26]. In general, we observed a shift to higher P_Y_ values for polymeric excipients by using the “density under pressure” approach for calculation. Similar observations were made for Starch1500^®^ by Schlack [23], and for Avicel by Krumme et al. [25] and Krumme [52]. An explanation was provided by Sonnergaard [53], demonstrating that the derived Heckel parameters such as yield pressure are predominantly influenced by the particle density; the higher the density value used for calculation, the higher the respective yield pressures. It was stated that there might be an influence on P_Y_ based on the particle density value per se. 

Based on the observations made in the current study, the question arises if the Heckel equation is applicable for polymeric excipients or, in general, for excipients showing densification under pressure above particle density. It should be considered in defining a common compaction pressure limit for the applicability of the Heckel analysis for excipients showing exceeding particle density might be reasonable. In addition, it should be discussed whether the porosity calculation for compression analysis should be adapted by means of the “density under pressure” in such cases. Moreover, if comparing different kinds of excipients, e.g., polymeric with nonpolymeric ones, whether it makes sense to use the same porosity calculation approach or if it should be tailored for each excipient based on the excipient’s properties; “density under pressure” for excipients exceeding the particle density and particle density for excipients showing no exceeding. 

All this leads to the ultimate question: what benefit remains for the Heckel analysis in the development of solid oral dosage forms if it requires a complex assessment of its applicability considering its limitations combined with a high error susceptibility in determining related measurands such as density? For us, it is certainly conceivable to use the particle density threshold instead. The threshold value can be assessed easily using any instrumented tablet press, providing knowledge of the compaction pressure above which elastic deformation occurs predominantly during compression. Knowing the threshold compaction pressure offers the opportunity to rate the risk of tablet defects caused by the stored elastic energy during formulation and process development.

## 5. Conclusions

The present study revealed that during compression of polymeric excipients, the particle density was already exceeded at low compaction pressures (CPs). In comparison, the particle density of compressed nonpolymeric excipients was reached either at higher CPs or never. We found that the threshold for this exceedance correlated with the start of a linear increase in elastic recovery (in-die). This means that the energy needed to achieve higher densification than the particle density was directly linked to the elastic deformation of the material itself (solid-state compression). Consequently, the threshold exceedance during tableting might increase the risk for tablet defects due to stored elastic energy and thus, should be avoided. Similar trends were seen for a model amorphous solid dispersion (ASD) containing ritonavir as a drug substance and copovidone as a matrix polymer. However, it was observed that the addition of a brittle filler (DI-CAFOS^®^A60) led to a threshold shift towards higher CPs, reducing the risk for tablet defects and increasing the design space for the compression process during development. 

In addition, the common Heckel compression analysis was shown not to be valid at high CPs, considering the particle density for calculation. In general, it might be questioned if the Heckel analysis is useful for polymeric excipients.

To conclude, the knowledge about the pressure threshold, where the density under compression exceeds the particle density during compression, could reduce the risk in tablet development as suitable fillers might be selected to either compensate for pronounced elasticity or assign safe pressure windows for production. 

## Figures and Tables

**Table 1:** Results of density measurements (ρ_pre_ and ρ_par_) and the resulting density ratio (ρ_ratio_).

Material	CP for ρ_pre_ [MPa]	ρ_pre_ [g/cm^3^]	ρ_par_ [g/cm^3^]	ρ_ratio_ [%]
Copovidone	503.0 ± 7.4	1.308 ± 0.026	1.207 ± 0.004	8.37
HPMC AS	505.9 ± 23.2	1.390 ± 0.004	1.281 ± 0.001	8.51
Soluplus^®^	500.0 ± 12.0	1.291 ± 0.004	1.176 ± 0.003	9.78
Starch1500^®^	507.6 ± 13.3	1.605 ± 0.005	1.490 ± 0.004	7.72
Avicel^®^PH101	500.8 ± 8.7	1.631 ± 0.005	1.558 ± 0.002	4.69
DI-CAFOS^®^A60	506.9 ± 17.7	2.407 ± 0.016	2.819 ± 0.001	−14.62
TriCaCi	501.7 ± 5.7	1.895 ± 0.011	1.967 ± 0.005	−3.66
FlowLac^®^100	515.1 ± 8.3	1.585 ± 0.003	1.546 ± 0.005	2.52
Pearlitol^®^100SD	509.8 ± 5.7	1.528 ± 0.003	1.466 ± 0.001	4.23
HME ASD	498.6 ± 5.6	1.281 ± 0.002	1.201 ± 0.001	6.66
HME TB	507.2 ± 10.9	1.386 ± 0.003	1.299 ± 0.001	6.70

**Table 2:** Results of calculated elastic modulus (E_mod_) based on ER_in-die_ linear equation (n.a. = not applicable).

Material	$a_{{\mathrm{ER}}_{\mathrm{eq}}}$	E_mod_ (GPa)
Copovidone	0.01635	6.1
HPMC AS	0.01554	6.4
Soluplus^®^	0.01731	5.8
Starch1500^®^	0.01471	6.8
Avicel^®^PH101	0.01124	8.9
DI-CAFOS^®^A60	n.a.	n.a.
TriCaCi	n.a.	n.a.
FlowLac^®^100	n.a.	n.a.
Pearlitol^®^100SD	0.00986	10.2
HME ASD	0.01538	6.5
HME TB	0.01353	7.4

**Table 3:** Calculated parameters from the Heckel analysis for all excipients and HME ASD and TB compressed at 100 MPa and 300 MPa (n.a. = not applicable, no sufficient linearity).

**COMPACTION PRESSURE AT 100 MPa**								
	**Calculated with Particle Density (ρ_par_)**				**Calculated with Density under Pressure (ρ_pre_)**			
	**Slope k**	**Intercept A**	**Mean Yield Pressure P_Y_ [MPa]**	**SF Corresponding to A**	**Slope k**	**Intercept A**	**Mean Yield Pressure P_Y_ [MPa]**	**SF Corresponding to A**
Copovidone	0.012 ± 0.000	0.603 ± 0.001	80.2 ± 0.2	0.453 ± 0.001	0.010 ± 0.000	0.559 ± 0.001	100.3 ± 0.3	0.428 ± 0.001
HPMC AS	0.024 ± 0.000	0.486 ± 0.006	41.8 ± 0.3	0.385 ± 0.004	0.014 ± 0.000	0.677 ± 0.026	69.1 ±1.8	0.492 ± 0.013
Soluplus^®^	0.018 ± 0.000	0.816 ± 0.007	57.1 ± 0.5	0.558 ± 0.003	0.013 ± 0.000	0.713 ± 0.021	75.0 ± 2.2	0.510 ± 0.010
Starch1500^®^	0.012 ± 0.000	0.875 ± 0.002	82.3 ± 0.6	0.583 ± 0.001	0.009 ± 0.000	0.798 ± 0.002	107.1 ± 0.8	0.550 ± 0.001
Avicel^®^PH101	0.015 ± 0.000	0.544 ± 0.005	65.0 ± 0.8	0.420 ± 0.003	0.012 ± 0.000	0.587 ± 0.008	80.8 ± 1.1	0.444 ± 0.005
DI-CAFOS^®^A60	0.003 ± 0.000	0.776 ± 0.038	293.2 ± 10.4	0.540 ± 0.017	0.006 ± 0.000	0.956 ± 0.007	181.2 ± 3.2	0.616 ± 0.003
TriCaCi	0.004 ± 0.000	0.682 ± 0.006	243.4 ± 2.7	0.494 ± 0.003	0.005 ± 0.000	0.714 ± 0.006	218.9 ± 2.3	0.510 ± 0.003
FlowLac^®^100	0.007 ± 0.000	0.873 ± 0.007	137.7 ± 6.0	0.582 ± 0.003	0.007 ± 0.000	0.852 ± 0.007	152.6 ± 6.5	0.573 ± 0.003
Pearlitol^®^100SD	0.008 ± 0.000	0.865 ± 0.008	129.7 ± 2.9	0.579 ± 0.003	0.007 ± 0.000	0.832 ± 0.008	153.6 ± 3.5	0.565 ± 0.003
HME ASD	0.013 ± 0.000	1.158 ± 0.005	78.8 ± 0.3	0.686 ± 0.001	0.009 ± 0.000	1.072 ± 0.006	111.6 ± 0.8	0.658 ± 0.002
HME TB	0.011 ± 0.000	1.218 ± 0.007	87.5 ± 2.6	0.704 ± 0.002	0.008 ± 0.000	1.125 ± 0.006	127.1 ± 3.8	0.675 ± 0.002
**COMPACTION PRESSURE AT 300 MPa**								
	**Calculated with Particle Density (ρ_par_)**				**Calculated with Density under Pressure (ρ_pre_)**			
	**Slope k**	**Intercept A**	**Mean Yield Pressure P_Y_ [MPa]**	**SF Corresponding to A**	**Slope k**	**Intercept A**	**Mean Yield Pressure P_Y_ [MPa]**	**SF Corresponding to A**
Copovidone	0.013 ± 0.001	0.595 ± 0.006	79.0 ± 3.2	0.448 ±0.003	0.009 ± 0.000	0.579 ± 0.006	106.4 ± 4.1	0.440 ± 0.004
HPMC AS	n.a.				0.007 ± 0.000	1.579 ± 0.053	140.3 ±7.3	0.794 ± 0.011
Soluplus^®^	n.a.				0.013 ± 0.000	0.712 ± 0.009	79.0 ± 0.4	0.509 ± 0.005
Starch1500^®^	0.010 ± 0.000	0.877 ± 0.001	101.4 ± 0.7	0.584 ± 0.001	0.007 ± 0.000	0.798 ± 0.007	134.8 ± 2.1	0.550 ± 0.003
Avicel^®^PH101	0.014 ± 0.000	0.572 ± 0.005	69.3 ± 0.7	0.436 ± 0.003	0.008 ± 0.000	1.030 ± 0.071	120.6 ± 6.1	0.642 ± 0.025
DI-CAFOS^®^A60	0.002 ± 0.000	0.943 ± 0.008	584.8 ± 62.0	0.610 ± 0.003	0.004 ± 0.000	1.083 ± 0.022	253.9 ± 32.7	0.661 ± 0.008
TriCaCi	0.003 ± 0.000	0.748 ± 0.002	336.0 ± 5.2	0.527 ± 0.001	0.003 ± 0.000	0.770 ± 0.002	290.9 ± 4.8	0.537 ± 0.001
FlowLac^®^100	0.006 ± 0.000	0.971 ± 0.002	159.5 ± 2.8	0.621 ± 0.001	0.005 ± 0.000	1.012 ± 0.006	194.9 ± 2.6	0.637 ± 0.002
Pearlitol^®^100SD	0.006 ± 0.000	0.945 ± 0.004	160.0 ± 4.7	0.611 ± 0.001	0.005 ± 0.000	0.978 ± 0.002	213.4 ± 6.2	0.624 ± 0.001
HME ASD	n.a.				0.009 ± 0.000	1.129 ± 0.008	115.5 ± 1.2	0.677 ± 0.002
HME TB	0.011 ± 0.000	1.163 ± 0.004	87.9 ± 2.8	0.687 ± 0.001	0.007 ± 0.000	1.117 ± 0.001	136.4 ± 4.7	0.673 ± 0.000

## Data Availability

The data presented in this study are available in the research article.
